# Exercise and Physical Therapy Interventions for Children with Ataxia: A Systematic Review

**DOI:** 10.1007/s12311-019-01063-z

**Published:** 2019-08-07

**Authors:** Helen Hartley, Elizabeth Cassidy, Lisa Bunn, Ram Kumar, Barry Pizer, Steven Lane, Bernie Carter

**Affiliations:** 1 Physiotherapy Department, Alder Hey Children’s NHS Foundation Trust, Eaton Road, Liverpool, L12 2AP UK; 2 Physiotherapy Department, Lunex International University of Health, Exercise and Sports, Differdange, Luxembourg; 3 School of Health Professions, University of Plymouth, Plymouth, UK; 4 Neurology Department, Alder Hey Children’s NHS Foundation Trust, Eaton Road, Liverpool, L12 2AP UK; 5 Oncology Department, Alder Hey Children’s NHS Foundation Trust, Eaton Road, Liverpool, L12 2AP UK; 6 University of Liverpool, Liverpool, UK; 7 Edge Hill University, Ormskirk, UK

**Keywords:** Exercise, Physical therapy, Paediatrics, Ataxia, Systematic review

## Abstract

The effectiveness of exercise and physical therapy for children with ataxia is poorly understood. The aim of this systematic review was to critically evaluate the range, scope and methodological quality of studies investigating the effectiveness of exercise and physical therapy interventions for children with ataxia. The following databases were searched: AMED, CENTRAL, CDSR, CINAHL, ClinicalTrials.gov, EMBASE, Ovid MEDLINE, PEDro and Web of Science. No limits were placed on language, type of study or year of publication. Two reviewers independently determined whether the studies met the inclusion criteria, extracted all relevant outcomes, and conducted methodological quality assessments. A total of 1988 studies were identified, and 124 full texts were screened. Twenty studies were included in the review. A total of 40 children (aged 5–18 years) with ataxia as a primary impairment participated in the included studies. Data were able to be extracted from eleven studies with a total of 21 children (aged 5–18 years), with a range of cerebellar pathology. The studies reported promising results but were of low methodological quality (no RCTs), used small sample sizes and were heterogeneous in terms of interventions, participants and outcomes. No firm conclusions can be made about the effectiveness of exercise and physical therapy for children with ataxia. There is a need for further high-quality child-centred research.

**Electronic supplementary material:**

The online version of this article (10.1007/s12311-019-01063-z) contains supplementary material, which is available to authorized users.

## Introduction

Ataxia is a common childhood movement disorder, with an estimated worldwide prevalence of 26/100,000 for both genetic and acquired causes [1]. Ataxia is most frequently caused by damage to or dysfunction of the cerebellum and its associated connections, and this is termed cerebellar ataxia. The primary features of cerebellar ataxia include reduced limb coordination (for example, dysmetria and tremor), postural and gait deficits, problems with oculomotor control and dysarthria [2]. Sensory ataxia refers to dysfunction of the proprioceptive input from the periphery and the ascending systems [3]. Sensory ataxia can disrupt limb co-ordination and, particularly, gait, depending on the site and size of lesion [4]. Ataxia may result in a range of functional difficulties involving balance and walking, reaching, grasping and manipulation, eye movement, swallowing and speech intelligibility [5, 6]. Childhood ataxias may be acquired (e.g. following stroke, traumatic brain injury (TBI), cerebral palsy (CP), cerebellar tumour), inherited (e.g. spino-cerebellar ataxia (SCA), Friedreich’s ataxia (FRA)) or idiopathic [2]. In the absence of effective pharmacological options, rehabilitation, particularly, physical therapy, remains the mainstay of treatment [7–9].

Eight literature reviews have previously reported on the effectiveness of rehabilitation (typically focused on physical therapy and exercise interventions) for primarily adults with ataxia [10–17]. A detailed analysis of these eight reviews was undertaken to determine whether a new systematic review that focussed exclusively on children with ataxia would add to knowledge to this field. The results of this analysis indicated that none of the reviews comprehensively searched for studies that included children or clearly reported the effect of interventions on children. Five reviews identified either one [12, 14, 16, 17] or two [15] studies that involved children; one review included one study that involved participants aged 17–69 years [11]; one review did not identify any studies involving children [13]; and one expressly focussed on adults [10]. None of the reviews that identified studies involving children discussed the results separately from studies involving adults. Furthermore, five reviews only included studies about people with a degenerative ataxia [11–13, 16, 17], and three reviews included people with ataxia presenting as part of another condition such as multiple sclerosis [10, 14, 15].

Studies involving children and young people with ataxia may have been omitted from these published reviews due to limitations in the search methods and restrictions placed on inclusion criteria which varied considerably (Table 1).

**Table 1:** Limitations of existing reviews of the evidence

Study	Limitation
Marquer et al. [15]	Narrative review, no clear search date or search strategy. Focussed on describing the assessment and treatment of postural disorders.
Synofzik and Ilg et al. [16]	Included only prospective studies using high-intensity training schedules and outcomes addressing gait and stance.
Trujillo-Martin et al. [11]	Included only studies with a minimum of three participants and a minimum 6-month follow-up period.
Martins et al. [13]	Included only studies published since 2000 and which scored at least five out of ten on the Physiotherapy Evidence Database Scale (www.pedro.org.au/).
Artigas et al. [12]	Used broad search terms but did not report inclusion criteria.
Fonteyn et al. [14]	Children were included but only prospective clinical trials, and case studies were included in the review if at least two different studies used the same intervention.
Milne et al. [17]	Included children and prospective and retrospective studies of randomised and non-randomised controlled studies and cohort studies, but not case studies or case series

Concerns about the lack of high-quality studies were raised by the review authors, but, overall, emerging evidence of the effectiveness of rehabilitation interventions was reported for adults living with a progressive ataxia [12, 13, 16, 17], and other causes of ataxia [10, 14, 15]. However, interventions that show promise with adults will not necessarily have the same impact with children and young people for several reasons.

Brain development continues throughout childhood as increasingly more sophisticated movement repertoires are acquired through experience-based learning [18]. Normative data derived from the International Cooperative Ataxia Rating Scale (ICARS) (a scale which quantifies the level of ataxia impairment), for example, has shown that typically developing children only approach their “adult norm” score of zero (indicating no coordination problems) at approximately 12 years of age [19]. Children’s central nervous systems may therefore respond differently to rehabilitation interventions when compared to a mature but similarly impaired adult system. Age is likely to affect engagement and compliance with the chosen modality or intervention and may impact the targeting and timing of rehabilitation efforts. Children have different information-processing capacities compared to adults and respond differently to motor learning and skill-acquisition paradigms, suggesting children may require more exercise practice time before learning is consolidated when compared to adults [20]. Certain cerebellar pathologies are more prevalent in children, e.g. midline floor of the fourth ventricle tumours are common in children, whereas many of the SCAs emerge only in adulthood. These different pathologies impact cerebellar function in different ways and may require different rehabilitation strategies. As none of the review authors searched specifically for studies with children, or focussed on, or reported interventions or outcomes for children and young people with ataxia, the overall picture of research in this field is not well understood. An up-to-date and comprehensive assessment of the evidence is required to develop a better understanding of, firstly, the effectiveness of exercise and physical therapy interventions for children and young people with ataxia and, secondly, the different types of interventions that have been investigated to date.

### Description of the Interventions

Exercise is defined as “physical activity that is planned, structured, repetitive, and purposive in the sense that improvement or maintenance of one or more components of physical fitness is an objective” (p. 128) [21]. Exercise may improve the following components of physical fitness: muscle strength, muscle endurance and cardiorespiratory fitness. Exercise interventions may be categorised as resistance training or aerobic (endurance) training based on the component of fitness the exercise programme is targeting. Resistance (strength) training is defined as the body’s muscles working or holding against an applied force. Body weight, free weights, machine weights and elastic bands may be used to apply force [22]. Aerobic training comprises the body’s large muscle groups moving in a rhythmic manner over a sustained period of time [22]. Examples of aerobic exercise include walking, running, cycling and arm ergometry. Endurance training or cardiovascular training is a type of aerobic training that includes activities that increase breathing and heart rates. Exercise programmes may target muscle strength, muscle endurance or cardiorespiratory fitness or a combination of these components described as “mixed training” [23].

Physical therapy aims to restore movement and function following injury, illness or disability using movement, exercise and manual therapy, as well as education and advice [24]. Physical therapy may include exercises as described previously and/or the following: task-specific training with the aim of (re)acquiring a motor skill (with or without using robotic exoskeletons); exercises that focus on regaining or sustaining control of the proximal muscles of the trunk, shoulder and pelvic girdle; exercises that aim to improve static and dynamic balance and proprioception as a component of postural control; and stretching exercises that aim to improve range of movement. Adjuncts, such as treadmill training with or without partial body weight support, functional electrical stimulation of voluntary muscles and exergames that use computer technologies to provide an interactive environment which requires limb movement to react to on screen game play (e.g. Wii, X Box), may also be included as part of a physical therapy training programme.

### Neuroscientific and Theoretical Foundations for Interventions

As part of a distributed system, the cerebellum plays a key role in motor control and motor learning [25, 26], and, for this reason, it was customary to believe that interventions to improve motor function for people with ataxia would be ineffective [27]. Recent evidence suggests that although adaptive learning is affected by cerebellar damage [28], motor learning is possible despite cerebellar pathology [29, 30]. Sparing of the deep cerebellar nuclei and the extracerebellar systems is thought to be a factor in recovery of motor function in children following cerebellar injury [28].

Contemporary rehabilitation approaches for people with cerebellar dysfunction may involve strategies that compensate for the underlying impairment, e.g. increasing inertia by weighting equipment, such as walking aids, or weighting the ataxic limb or strategies that aim to improve or restore function by treating cerebellar-specific impairments, e.g. through balance and ocular training [31]. The potential mechanisms underlying the restorative and compensatory approaches are the subject of ongoing investigations (see, for example, Bhanpuri et al. [32]). It is also possible that exercise interventions, as defined previously, may increase physical fitness and physical activity levels and deliver health-promoting effects. Exercise interventions may also confer benefits that reside outside of the biomedical sphere by having a positive effect on a child’s well-being and life experience. These broader outcomes are considered essential to understanding childhood disability and should be incorporated in research protocols [33].

The aims of this systematic review were to map and critically evaluate the type, range, scope and scientific quality of exercise and physical therapy interventions on impairment, function, participation and quality of life for children and young people with ataxia. The results of this review aim to inform healthcare professionals about the effectiveness and quality of the evidence for these interventions and to assist the development of future research in this field.

## Methods

The PICO (population, intervention, comparisons and outcomes) framework was used to develop the literature search strategy.

### Types of Studies

All prospective and retrospective intervention studies where before and after outcome data were collected, such as randomised controlled trials, quasi-randomised controlled trials, non-randomised studies and single-case experimental designs, were included. Case studies were included if measures of outcome were reported. Case reports and case descriptions where the impact of an intervention was not determined, and where no measures of outcome were reported, were excluded from the review.

### Participants

Children and young people 18 years old or under, of any functional ability, with ataxia as the primary impairment were eligible. Studies that included participants who were under 18 years as well as those over 18 years of age were categorised as “mixed aged group” studies and were included in the review but reported separately. If data from participants at or under 18 years old could be extracted from these “mixed age group” studies, these data were reported separately.

Participants with ataxia as a result of posterior fossa tumour, stroke, CP, brain injury, idiopathic cerebellar ataxia, autosomal-recessive ataxia (e.g. FRA; early-onset ataxia, such as ataxia telangiectasia (AT); adolescent-onset ataxia) or autosomal-dominant ataxia were included. Studies where participants had other childhood conditions, where ataxia is a feature but is not the primary motor impairment (e.g. Angelman’s syndrome, Wilson’s disease), were excluded. Participants with other conditions known to affect the cerebellum but with other primary signs and symptoms, such as developmental coordination disorder and autism, were also excluded. Studies that included participants with ataxia as a result of self-limiting conditions that usually resolve (e.g. some types of acute neurotoxicity or infection) were excluded.

### Types of Interventions

Studies using or describing the following exercise, training and physical therapy interventions were included:

- Exercise interventions that aimed to improve one of the following components of physical fitness, i.e. muscle strength and/or muscle endurance and/or cardiorespiratory fitness and may include, for example, resistance training and/or aerobic training exercises
- Physical therapy interventions that aimed to improve co-ordination and/or dexterity and/or balance and/or posture
- Exercise interventions or physical therapy interventions that used exercise devices, such as treadmills, body weight support systems and robot-assisted exercise protocols to improve a component of physical fitness and/or co-ordination and/or dexterity and/or balance and/or posture
- Exercise interventions or physical therapy interventions that involved riding horses or mechanical horses, exercises in water, including swimming, to improve a component of physical fitness and/or co-ordination, and/or dexterity and/or balance and/or posture
- Physical therapy interventions that aimed to improve physical functioning through task- or part task-specific practice, e.g. constraint-induced movement therapy (CIMT)
- Physical therapy interventions described as “Bobath” or neurodevelopmental therapy (NDT)
- Functional electrical stimulation (FES) and/or neuromuscular electrical stimulation (NMES) and functional orthoses, such as Lycra garments, and upper and lower limb splints, were only included if the intervention was used in conjunction with exercise interventions or physical therapy interventions (reflecting conventional practice) or as a comparison to exercise interventions, as defined previously, to improve one of the components of physical fitness or co-ordination, dexterity, balance, posture or function.

The following interventions were excluded because they were not considered to be exercise or physical therapy interventions: psychological interventions, interventions restricted to improving communication (speech or other means of communication) or swallowing, breathing exercises, acupuncture, vibration therapy or types of non-invasive brain stimulation (in isolation or combined with exercise interventions).

Comparisons of interest (where study design permitted) were exercise and physical therapy interventions (as described previously) versus no treatment, or usual care, or a comparison of one exercise or physical therapy intervention with another exercise or physical therapy intervention.

### Outcome Measures

As there are no gold standard outcome measures for children with cerebellar ataxia, the following outcomes were indicative and not specified as inclusion criteria for this review.

#### Primary Outcomes

1. Activity defined as a person’s ability to execute a task [34]. Primary outcomes may focus on completing activities of daily living and application of skills within a range of different settings (e.g. the community/home/school/primary or secondary care setting). For example, the Gross Motor Function Measure [35] and WeeFIM [36].
2. Participation defined as a person’s involvement in a life situation [34]. For example, the Paediatric Evaluation of Disability Inventory [37].
3. Health-Related Quality of Life (HRQoL) defined as the impact of disease and treatment on physical, psychological and social domains of health as distinct areas that are influenced by a person’s experience, beliefs, expectations and perceptions [38, 39]. For example, the Child Health Questionnaire [40]. The incidence and nature of adverse events, such as injury and delayed-onset muscle soreness, where reported.

#### Secondary Outcomes

Body functions and body structures defined as changes in physiological systems or in anatomical structures [34], for example, muscle strength, endurance, fatigue, pain, cardiorespiratory fitness, balance, ataxia severity and coordination. For example, the Scale for the Assessment and Rating of Ataxia [41].

Any measure that purported to measure these outcomes was included, regardless of whether or not it was validated specifically for children with ataxia.

Outcomes were collected for the following time points: short term (0 to 1 month post-intervention), intermediate term (> 1 month to 6 months post-intervention), and long term (> 6 months post-intervention).

### Search Methods for Identification of Studies

The following databases were searched from inception to February 2018: Allied and Complementary Medicine Database (AMED), Cochrane Central Register of Controlled Trials (CENTRAL), Cochrane Database of Systematic Reviews (CDSR), CINAHL (EBSCOhost), ClinicalTrials.gov, EMBASE (OVID), Ovid MEDLINE, Physiotherapy Evidence Database (PEDro) and Web of Science (all databases). The conference proceedings of the International Society for Paediatric Oncology (SIOP), the International Symposium on Pediatric Neuro-oncology (ISPNO) (2005–current) and the World Confederation for Physical Therapy (WCPT) were also included.

The search terms child* OR pediatric OR paediatric OR adolescent OR infant were combined with results from a second search for the terms ataxi* OR atax* OR co-ordination OR “motor impairment” OR “balance impairment” OR “postural instability”, and these results were combined with results from the third search for “physical therapy” OR “physiotherapy” OR “rehabilitation” OR exercise OR “exercise therapy” OR “physical activity” OR “home exercise programme” OR “balance training” OR “postural training” OR “co-ordinative training” OR “hydrotherapy” OR “aquatic therapy” OR “neurodevelopmental therapy” OR “strength training” OR “muscle strengthening” OR “virtual training” OR “treadmill training” OR “kinesiology taping” OR “lycra”. This search strategy was adapted as appropriate for each source. Limits were not imposed on searches for language, date or publication status. The reference lists of included studies and relevant systematic reviews identified with the search results were also searched.

### Selection of Studies

Two review authors (HH and EC) independently screened the titles and abstracts of the search results and excluded studies that did not meet the search criteria. Where studies appeared to meet the inclusion criteria, or where there was any doubt about inclusion, the full text of the published paper was retrieved. Two review authors (HH and EC) independently reviewed these papers against the inclusion criteria. Any disagreements regarding the exclusion of studies, at any stage of the review process, were resolved through discussion. Where an agreement about inclusion or exclusion could not be reached, a third review author (LB) made the final decision.

### Data Extraction and Management

Two review authors (HH and EC) extracted data independently. Disagreements about the extraction of data were resolved by discussion. If a resolution was not reached, a third review author was consulted (LB). (The data extraction checklist is available as Supplementary material). The following information was extracted where possible:

- Authors, title, abstract, publication type, publication record, country of origin
- Study design
- Sample size
- Study population: sex, age, ethnicity, diagnosis, type of ataxia and gross motor function, where sufficient information was provided. Walking function was recorded, where possible, as unaided walking, walking with aids or unable to walk, and according to other validated measures, e.g. Gillette Functional Assessment Questionnaire [42]. Ataxia severity was recorded where possible, e.g. Scale for the Assessment and Rating of Ataxia (SARA) [41] and Brief Ataxia Rating Scale (BARS) [43].
- Intervention: aim of the intervention, type of exercise programme (e.g. aerobic exercise), mode of delivery (e.g. home programme), type(s) of location(s) where the intervention occurred (including any necessary infrastructure or relevant features), supervised or unsupervised programme, exercise mode (e.g. cycle ergometry, treadmill), exercise dose (i.e. duration, intensity, and frequency of exercise), tailoring/modification of the intervention to an individual (what, why, when, how), duration of programme.
- Intervention provider: profession, expertise, background, specific training received.
- Compliance: fidelity (whether the intervention was delivered as intended) and adherence to the prescribed dose (frequency, intensity, duration); how and by whom this was assessed.
- Outcome measures.
- Results: short term (0 to 1-month post-intervention), intermediate (greater than 1 month to 6 months post-intervention), and long term (> 6 months post-intervention) follow-up.
- Adverse effects.
- Conflicts of interest, declarations of conflicts of interest and sources of funding.

The methodological quality of the included studies was appraised using the Oxford Centre for Evidence-Based Medicine (OCEBM) level of evidence classification [44] (Table 2). This appraisal method is consistent with other internationally recognised guides. Where disagreements could not be resolved through discussion between HH and EC, a final decision was made by a third author (LB).

**Table 2:** Oxford Centre for Evidence-Based Medicine 2011 levels of evidence

Level of evidence	
Level 1^a^	Systematic review of randomised trials or *n*-of-1^b^ trials
Level 2^a^	Randomised trial or observational study with dramatic effect* (*level may be graded down on the basis of study quality, imprecision, indirectness, etc.)
Level 3^a^	Non-randomised controlled cohort or follow-up study
Level 4^a^	Case series, case–control studies or historically controlled studies.
Level 5	Mechanism-based reasoning

^a^Level may be graded down on the basis of study quality, imprecision, indirectness, inconsistency between studies, or because the absolute effect size is very small; level may be graded up if there is a large or very large effect size.

^b^Definition of *n*-of-1 trial: a variation of a randomised controlled trial in which a sequence of alternative treatment regimens is randomly allocated to a patient. The outcomes of regimens are compared, with the aim of deciding on the optimum regimen for the patient

## Results

### Type, Range, Scope and Methodological Quality of Selected Studies

**Fig. 1:**
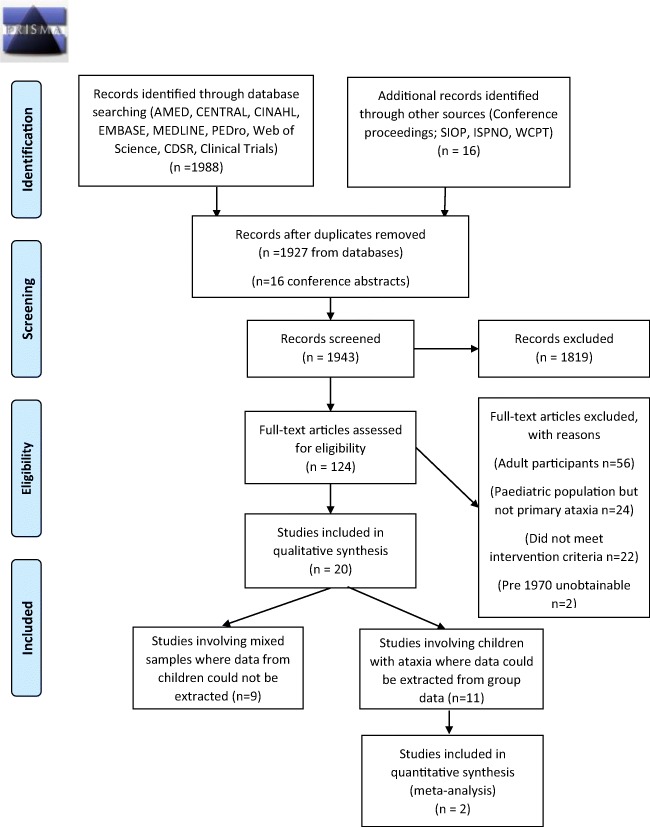
PRISMA flow diagram: search results

After the removal of duplicates, 1927 studies and 16 conference abstracts were screened. Following screening, 124 full-text studies were assessed for eligibility. Of these, 56 were excluded as they did not involve children, 24 did not have ataxia as the primary diagnosis/presenting feature and 22 did not meet the intervention criteria stated in the search strategy. Two studies could not be obtained [45, 46]. Twenty studies were included in this review. All studies were published in the last 20 years (1999–2017), with ten in the last five years. The PRISMA flow diagram [47] is presented in Fig. 1.

A total of 40 children with ataxia as a primary impairment participated in the studies included in this review. Where stated, the ages of the children with ataxia ranged from 5 to 18 years (median 13 years). Where stated, the duration of the intervention ranged from 2 weeks [48] to 19 months [49] (median 7 weeks) and intensity ranged from 10 min [48] to 2 h per session [50] (median 45 min per session). Frequency ranged from once every three months [51] to six days per week [48] (median 3 sessions a week), excluding an outlier where Lycra garments were prescribed daily for six weeks, for 6 h a day plus usual physical therapy care for 10–30 min per day [52].

### Studies Involving Mixed Groups Where Data from Children with Ataxia Could Not Be Extracted

Nine of the included studies comprised mixed groups of participants, either children with adults or children with ataxia with children with other primary impairments. Data from the children with ataxia in these studies could not be extracted for this review. Five studies with children with cerebral palsy, with sample sizes ranging from 8 to 70 participants, included one [50, 53], two [54], three [55] or six [56] participants with ataxia as their primary impairment. The methodological quality of these studies was judged at OCEBM level 3 for an RCT [50] downgraded from level 2 due to increased risk of bias for being underpowered and OCEBM level 4 for four single group (before and after) studies [53–56]. The results from participants with ataxia in these studies were not reported separately from the group results, and, therefore, the data were not able to be extracted or included in this review. Interventions included the following: NDT vs Adeli Suit Treatment (training of gross motor function whilst wearing an externally fitted suit which provided stability and resistance) [50], strength training [53], NDT [54], aerobic treadmill training [55] and robot-assisted gait training [56]. Biffi et al. [57] conducted an OCEBM level 4 before and after trial, to investigate the efficacy of an immersive virtual reality platform to enhance walking ability in children with acquired brain injury. One child with ataxia was included in a total sample of 12 children. Significant improvements were reported in gross motor function, endurance (6MWT) and autonomy in daily life. Overall, small and predominantly short-term benefits were reported in this group of studies which cannot be used to draw conclusions about the effectiveness of these interventions for participants with ataxia.

Of the remaining three studies in this group, Nardone et al. [58] included one young person aged 16 in an otherwise adult sample of 27 participants with cerebellar dysfunction caused by either degenerative disease or cerebellar stroke, in an OCEBM level 4, single group (before and after) study. Small short-term positive effects in both groups were reported on body sway and gait parameters and the FIM (Functional Independence Measure) following a balance and gait training protocol. Sabel et al. [59] conducted a randomised cross-over trial (downgraded from OCEBM level 2 to level 3 due to increased risk of bias for being underpowered) that compared active video gaming and coaching with usual care in a group of 13 children following treatment for brain tumour. Four of the cohort had posterior fossa tumours. The results demonstrated that the home-based intervention was feasible and improvements in body coordination were reported using the BOT2 (Bruininks-Oseretsky Test of Motor Proficiency). Santos et al. [60] included one child aged 15 years in an otherwise adult group of 28 people with SCA in an OCEBM level 4 prospective (before and after) feasibility trial of virtual reality balance training. Improvements were reported in balance and quality of life measured using the Berg Balance Scale, Dizziness Handicap Inventory and the SF-36 (Short-Form—36; a patient-reported outcome measure). None of the data for the children with ataxia in these studies were reported separately, and, therefore, no conclusions could be drawn about the effectiveness of the intervention for these participants.

### Studies Involving Children with Ataxia Whose Data Could Be Extracted

The remaining eleven studies (summarised in Table 3) involved children with ataxia whose data could be extracted for this review [48, 49, 51, 52, 61–67]. Schatton et al. [66] included data from one participant previously reported in the *n* = 1 pilot study conducted by Synofzik et al. [67]. In the following summary, data from this child have only been counted once. The studies included here were conducted mainly in North America (*n* = 4), with additional contributions from Australia, Brazil, Germany, New Zealand and the United Kingdom. This group of studies included a total of 21 children, aged 5 to 18 years; eleven boys and nine girls (one paper did not state gender), with progressive ataxia (*n* = 14), ataxic CP (*n* = 3), cerebellar/brain stem infarct (*n* = 1), traumatic brain injury (*n* = 1), cerebellar tumour (*n* = 1) or a non-progressive cerebellar ataxia (*n* = 1).

**Table 3:** Data extraction for the eleven main studies

Study	Study design	Participants				Intervention			Outcome measures	Results Short term (ST) Intermediate term (IT) Long term (LT)	Compliance (fidelity and adherence)	Adverse effects	Oxford
		Age/sex	Size	Diagnosis	Functional level	Description	Dose: duration, frequency, intensity	Provider/setting					
Ada et al. [48] Australia	SCED (ABA design)	5-year-old female	*N* = 1	Cerebellar tumour (low grade) resected 3.5 years previously.	Reported UE coordination problems.	Dexterity training using a computerised tracking task on a computer.	2/52, 12 sessions, 10′	Home, supervised by parents	Finger to nose test, 9HPT	ST: 8% improvement in tracking. FNT and 9HPT improved but not significantly. IT and LT not reported.	Reported good adherence to the intervention.	Reported as not harmful	4
Cernak et al. [61] USA	Case report	13-year-old female	*N* = 1	Cerebellar ataxia post-brain haemorrhage (16/12 previous).	Non-ambulatory.	Partial body weight-support treadmill training with over-ground practice.	4/52, 5×/wk, 40′. GAP 1/12. Then 4/12, 5×/wk, 30′	PT dept. and home-based training (with rehab assistant)	Gillette, Functional Walking Scale, WeeFIM (transfers and mobility subscale), number of unassisted steps.	ST: Minimal change at 1/12. IT: At 6/12 Gillette improved to walking for household distances. Transfers improved from moderate assistance to modified independence. Walking improved from maximum assistance to supervision. No. of unassisted steps improved from 0 to 200 LT not reported.	19/20 sessions completed in clinic. Not reported for home training	Fatigue and discomfort from harness	4
Da Silva and Iwabe-Marchese [62] Brazil	Case report	12-year-old male	*N* = 1	Ataxic CP	GMFCS level II	Video gaming targeted at balance using the Wii (with balance board).	4/12, 3×/wk 30′. Total 40 sessions	Not reported. Setting unclear.	GMFM-66, BBS, gait kinematics.	ST: BBS increased from 48 to 53 points, GMFM: no change in dimensions A–C; D increased from 64.63 to 65.33, dimension E increased from 72.63 to 81.98, the overall mean score improved from 71.69 to 77.46. Gait parameters: no change reported. IT and LT not reported	Not reported	Not reported	4
Frank et al. [63] USA	Case report	6-year-old female	*N* = 1	Ataxic cerebral palsy	Ambulatory GMFCS level 1	Hippotherapy	8/52, 2×/wk, 45′ (16 Rx sessions)	PT delivered Rx at the stables.	GMFM-66, PODCI, PSPCSAYC.	ST: GMFM 66, dimension D: no change (95) Dimension E improved from 87.5 to 93. PODCI improved significantly in 3 domains. PSPCSAYC scores on 2 of 4 domains improved by 2 points. IT: GMFM 66 D improved to 97.4, E improved to 94.4. PODCI improvement in 3 domains. PSPCSAYC minimal change. LT not reported	Number of sessions reported. HEP adherence reported.	Not reported	4
Harris-Love et al. [51] USA	Case report	14-year-old female	*N* = 1	FRA	Walking frame and powered wheelchair for mobility. Assistance of 1 to stand.	PT and adapted PE inc; bimanual task, task-orientated training, strengthening, stretching, gait training using a walking aid.	1×/month, 60′ (school) for 12/12, plus × 1/quarter 60′ (PT dept.), plus 20–30′ daily adapted PE, plus HEP, 5×/wk	PT dept., school and home	9HPT, SLST, manual muscle testing, passive ROM, gait speed, DLST, step length asymmetry, step time asymmetry, self-report falls history.	ST: at 12/12 9HPT reduced (60.0 to 56.6 s). ROM static or improved. MMT declined SLST increased 2.7 to 2.9. Fall rate decreased (12 to 3.) Gait speed varied depending on walker type. IT and LT not reported.	Not reported	Not reported	4
Ilg et al. [64] Germany	Before/after, no control group (intra-individual control design)	Age 11–20. 5 male, 5 female	*N* = 7/10 ≤ 18 years old	Children with spinocerebellar ataxia. 2–17 years post-diagnosis	SARA score 7–13.5	X Box coordinative training.	2/52, 4×/wk. 60′. Then, 6/52, varied intensity; 20–175′ per wk.	Lab-based training supervised, followed by home-based training.	SARA, Dynamic Gait Index (DGI), motion analysis (leg placement), ABC scale (balance confidence) measured at baseline, pre-treatment, post 2 weeks lab training, post 6 weeks home training.	ST: significant improvement in SARA (− 2 average) and DGI. Improvements in lateral sway and error during leg placement task. Non-significant improvements reported in ABC. IT and LT not reported.	Noted training intensity correlated with improvement in SARA posture subscore.	Not reported	3
Mulligan et al. [65] New Zealand	SCED (noted second intervention shorter) (ABCB design)	9 years old	*N* = 1	Non-progressive congenital ataxia.	Able to climb stairs without a rail. Modified TUGG (from the floor) at first assessment: 72 s.	Compared two PT interventions: Rx 1—strengthening pelvic/trunk musculature and practising midline in sitting and kneeling. Rx 2—challenge postural control in different positions with head mvts performed simultaneously to reduce amount of visual information.	11/52, 3×/wk, 30years GAP 5/52. Then 5/52, 3×/wk 30years	Rx 1: PT in school Rx 2: researcher, setting unclear	Modified TUGG GMFM, GMPM, timed independent stair climbing.	ST: mTUGG improvement of 35 s (from first intervention to 5/52 post end of 2nd intervention). GMFM overall improvement from 81 to 96% at end Rx 2. GMPM not clearly reported (graph compared to reported results). Timed stair climbing improvements reported with and without a rail. Reported better maintenance of results at end of second treatment block LT not reported	Not reported	Not reported	4
Nicholson et al. [52] UK	Before/after (measures on single occasion)	*N* = 12, ages 2–17. Male *n* = 7, female *n* = 5.	*N* = 1/12 child with ataxia	CP	Upper limb impairment	Lycra garment (continued to receive usual therapy during study period)	2 weeks initial gradual exposure, then 6 h per day for 6/52.	Mostly home setting (not supervised)	PEDI, reach and grasp (motion analysis), self-devised parent questionnaire re practicalities of the Lycra garment.	ST: improvements in PEDI self-care +8, mobility +4, social domains +7. No change in PEDI care giver assistance score. Improved trunk stability and upper limb function reported. Parental questionnaire not reported. IT and LT not reported.	Group but not individual daily use of the garment reported.	Impaired functional mobility, discomfort. Found uncomfortable to crawl in suit	4
Sartor-Glittenberg and Brickner [49] USA	Retrospective case report	*N* = 3 aged 16–22. One aged 16 with ataxia	*N* = 1 with ataxia	TBI (5/12 post).	Walked with a walking frame and maximum assistance of 2.	Mixed group PT and individual Rx. Activities to improve proximal stability, coordination and balance. Outpatient day programme. Also included climbing on an artificial wall in rock climbing gym.	77/52, 4-5×/ wk, weaned down to 1–2× wk.	Supervised with PT.	Muscle strength (0–5 scale), coordination (timed heel to shin, toe taps), BBS, SLST, FES, 6MWT, participation in activities via interview and observation.	ST: Increased lower limb strength, improved co-ordination in both LEs, BBS improved from 4 to 23, SLST improved from 0 to 3.5 s (R), 0 s to 1.5 s (L), FES improved from 37 to 95, 6MWT improved from 61 to 259 m. IT and LT not reported.	Not reported re therapy sessions, diary to HEP completed	Not reported	4
Schatton et al. [66] Germany	Before/after, no control group (intra-individual control design)	Age 6–29. 7 male, 3 female	*N* = 6/10 ≤ 18 years old	Children with SCA	SARA score 13–29	Exergame training. (Nintendo Wii® and Microsoft XBOX Kinect®)	Phase1; 1/52 lab, 4 × 60 min session then 5/52 at home. Phase2; 2/7 booster then 5/52 home training ×3 wk 45 min per session	Lab-based training supervised, followed by home-based training.	SARA, GAS, Romberg sitting task. Measured at baseline, pre-treatment, after phase 1, after phase 2.	ST: significant improvement in SARA (− 2.5 average). Higher GAS. Reduced body sway. IT and LT not reported.	Noted training intensity at home correlated with improvement in SARA	Not reported	3
Synofzik et al. [67] Germany	Case report	10-year-old male	*N* = 1	AT diagnosed at 3 years old	SARA score gait 7/8 (severe ataxia)	Video gaming coordinative training.	1/52 clinic, frequency and intensity not stated 2 update sessions. Then 5/52 home. Update then 6/52 home.	PT (lab-based) and home-based	SARA, GAS, sway in sitting.	ST: no change between 2 baseline phases. End of intervention SARA improvement of 4 points. GAS standing + 2, sitting + 1. Mvt analysis: less sway in sitting 2nd baseline to end of intervention. IT and LT not reported.	Not reported	Not reported	4

Abbreviations not appearing elsewhere: PE, physical education; HEP, home exercise programme; /52, per week; /12, per month; Mvt, movement; Rx, treatment; SLST, single-leg stance test; FES, falls efficacy scale; DLST, double-limb support time; LE, lower extremity; SCA, spinocerebellar ataxia; UE, upper extremity; Wk, week; AT, ataxia telangiectasia

Five studies provided balance, coordination or dexterity training [48, 62, 64, 66, 67]; three provided mixed training, classified as conventional physical therapy [49, 51, 65], one provided aerobic treadmill training [61], one provided horse-riding training [63] and one provided a full-body Lycra suit in addition to usual care [52].

The duration of the intervention ranged from 2 weeks [48] to 19 months [49] (median 12 weeks). Where stated, intensity ranged from 10 [48] to 60 min per session [51] (median 37.5 min per session). Frequency ranged from once every three months [51] to six days per week [48] (median 3 sessions a week), excluding Nicholson et al. [52], where Lycra garments were prescribed daily for six weeks, for 6 h a day plus usual physical therapy care for 10–30 min per day.

Nine of these studies were judged as OCEBM level 4 evidence; five single case reports [51, 61–63, 67], one case series [49], two single case experimental designs [48, 65] and one single group (before and after) design [52]. Two before and after studies with intra-individual comparison, blind assessment and extended baselines were elevated to OCEBM level 3 evidence [64, 66].

The Joanna Briggs Institute checklist for case reports was used to evaluate the quality of the case reports and the case series (*n* = 6) [68]. The two single case experimental designs (SCEDs) were evaluated using the CONSORT agreement for the reporting of *n* = 1 trial [69]. Studies categorised as before and after trials (*n* = 3) were evaluated using the NIH quality assessment tool for before–after (pre–post) studies with no control group [70]. The separate evaluation of the methodological quality of these eleven studies identified comparable strengths and limitations. For this reason, the results of the critical appraisal of this group of studies were considered together.

Characteristics, such as age, gender, diagnosis and genetic details (where relevant), were consistently reported but varied in the amount of detail offered. Psychosocial details were provided in one study [49]. Imaging results were reported by Sartor-Glittenberg and Brickner [49] and in supplemental information by Synofzik et al. [67]. Ataxia severity was rarely described in detail and only measured using a specific ataxia scale (SARA) in three studies from the same research laboratory [64, 66, 67]. Gross functional capacity was classified in four studies using the Gross Motor Function Classification System (GMFCS) [62, 63], the Gillette Functional Walking Scale and WeeFIM [61] or the Paediatric Evaluation of Disability Inventory (PEDI) [52]. Walking ability was described but not consistently measured in all relevant studies. Interventions were well-described, and the duration, frequency and intensity (dose) were consistently reported across all studies. Decisions about the prescribed dose of the intervention were not justified with respect to relevant theories or the results of other studies. Compliance was not consistently reported, particularly for home-based exercise programmes. Three studies identified primary outcomes [61, 64, 66]. Five studies reported measurement properties (validity and reliability) for one or more outcomes [49, 52, 61, 63, 65]. Only one study measured participation and quality of life outcomes [63]. All studies reported short-term outcomes (0–1 month post-intervention). No long-term outcomes were reported. Assessor blinding was reported in four studies [48, 64, 66, 67]. Adverse events were not routinely reported. One study reported that the intervention was not harmful [48], and one study clearly reported harmful effects [52]. None of the included studies reported involving children and/or parents in the design or evaluation of the intervention.

### Effectiveness of the Interventions

In the following evaluation, minimal detectable change (MDC) and minimal clinically important difference (MCID) scores have been provided where available to facilitate judgement of the reported effectiveness of interventions. Where paediatric data are not available, adult data have been used to provide proxy comparisons.

### Conventional Physical Therapy

Three papers reported the effects of conventional physical therapy. Harris-Love et al. [51] used bimanual task practice, task-orientated training, stretching, strengthening and gait training using walking aids with a 14-year-old child with FRA. The intervention was provided once every three months over a 12-month period (60 min per visit) and continued as a home exercise programme five times a week. Monthly school-based physical therapy continued (60 min per session) plus school-based physical education (20–30 min per session, up to five times per week). The additional intervention equated to an extra 4 h of hospital-based physical therapy plus the home exercise programme, five times per week over a 12-month period. The improvement of 3.4 s on the 9-hole peg test (9HPT) was not considered clinically meaningful; however, a reduction in the number of falls from 12 to 3 falls per month (self-report) in the context of a measured deterioration in areas such as strength and gait speed may be considered a clinically significant change for a child living with a progressive condition.

Mulligan et al. [65] used a SCED (ABCB) with a child with non-progressive congenital ataxia (severity not reported), comparing strengthening and balance training (30 min, three times a week for eleven weeks) with interventions aimed to challenge postural control (30 min, 3 times a week for five weeks). Improvements were reported in the modified Timed Get Up and Go (TUG), Gross Motor Function Measure (GMFM), stair climbing and the Gross Motor Performance Measure. However, it was difficult to evaluate the separate effects of each intervention as multiple measures were not undertaken in each phase, standard SCED statistical analysis was not used and trends could be observed in the data from the A phase into the other phases.

Sartor-Glittenberg and Brickner [49] reported a retrospective case report of a 16-year-old boy in the subacute phase following TBI. Ataxia severity was not measured but was reported as severe. He required a walking frame and the maximum assistance of two people to walk short distances. A wide range of interventions were provided during 187 therapy sessions over 19 months. Improvements were reported for all outcomes. An improvement of 19 points in the Berg Balance Scale (BBS) exceeded the MDC of 5 points relevant for older adult clinical populations with an initial score of 0–24 points [71]. An improvement of 198 m in the Six-Minute Walk Test (6MWT) exceeded the MCID reported as relevant for adults with a range of medical conditions [72]. Motor co-ordination improved but did not reach age-equivalent norms.

### Video Gaming and Computer-Assisted Training for Dexterity/Coordination and Balance

Five studies reported a positive effect of video gaming or computer-assisted training in children/young people with ataxia. As the participant in Synofzik et al. [67] was included in the data presented in Schatton et al. [66], only data from this second study are presented in this summary. Ada et al. [48] reported short-term but not statistically significant improvements in elbow dexterity (finger–nose test) and a timed upper limb task (9HPT) following a 2-week home programme of dexterity training for 10 min per day, using a computer-assisted elbow-tracking task (gravity eliminated), with a 5-year-old girl described as having severe upper limb ataxia following resection of a posterior fossa tumour. Da Silva and Iwabe-Marchese [62] reported immediate improvements following a 4-month programme of video game balance training (Nintendo Wii), in addition to usual care, for a 12-year-old boy with ataxic CP (GMFCS II—able to walk in most settings). A six-point improvement was reported in the GMFM-66 (exceeding the MCID for a large effect size reported by Oeffinger et al. [73]) and a five-point improvement in BBS (exceeding an MDC of four points relevant for older adults with an initial score of 45–56 points [71]). No improvement in gait kinematics was reported.

Ilg et al. [64] conducted an intra-individual control study using an eight-week video co-ordination-game training (X Box Kinect) programme (2 weeks in clinic (four 1-h training sessions) followed by 6 weeks at home) with 10 children and young adults (*n* = 7 ≤ 18 years old) with an inherited progressive ataxia as their primary impairment (mean SARA 10.9, range 7–13.5). A mean group change reflecting a 2-point improvement in SARA (more than one point change would be considered a MCID for adults with a progressive ataxia [74]) and improvements in sway and leg placement were reported. Schatton et al. [66] reported a mean 2.5-point improvement in SARA scores (exceeding the one point MCID SARA change considered relevant for adults with a progressive ataxia [74]), at the end of a 12-week (1 week in clinic, 5 weeks at home, two update sessions and a further 5 weeks at home) video gaming programme (Nintendo Wii) using whole body-controlled commercially available games for ten participants (*n* = 6 ≤ 18 years old) described as having advanced SCA.

As Ilg et al. [64] and Schatton et al. [66] used SARA as their primary outcome measure and provided data for all participants at all time points, data from these higher quality studies were pooled to conduct a meta-analysis of the effect of video game training on SARA scores. A comparison of change in SARA scores across time irrespective of age indicated a statistically significant and clinically meaningful reduction (improvement) in SARA scores from baseline to the end of the intervention (median reduction of 2 points, *p* < 0.001) (Table 4). A comparison of training time (overall dose) indicated that participants in Schatton et al. [66] spent a median of 160 min training compared to those in Ilg et al. [64] who spent a median of 70 min training (Table 5). This difference was statistically significant (*p* = 0.03), but the increased dose does not appear to have made a difference to outcome as measured by SARA, suggesting optimal dosages are yet to be determined. A comparison of change across time by age using pooled data from 13 children (≤ 18 years old) with pooled data from seven adults indicated that although SARA scores for children improved by a median of 0.5 points more than adults, the difference was not statistically significant (*p* = 0.49) (Table 6). Adults in these studies completed a median of 18 extra minutes of training compared to children, but the difference in training time was not statistically significant (*p* = 0.49) (Table 7).

**Table 4:** Comparison of change across time irrespective of age (*n* = 20)

	Time point 1	Time point 2	Significance
SARA median change over time (IQR)	13.5 (9.5)	11.5 (8.3)	*p* < 0.001^a^

^a^Wilcoxon signed-rank test

IQR, interquartile range

**Table 5:** Comparison of training time irrespective of age

	Schatton et al. [66]	Ilg et al. [64]	Significance
*N*	10	10	
Median time (IQR)	159.9 (23.3)	70.5 (110.5)	*p* = 0.03^a^

^a^Mann–Whitney *U* test

IQR, interquartile range

**Table 6:** Comparison of change across time by age

	Age 18 and under	Age 18 and over	Significance
*N*	13	7	
SARA median change over time (IQR)	2 (2.8)	1.5 (1.0)	*p* = 0.49^a^

^a^Mann–Whitney *U* test

IQR, interquartile range

**Table 7:** Training time (minutes)

	Age 18 and under	Age 18 and over	Significance
*N*	13	7	
Median time (IQR)	132 (122.4)	150 (45.0)	*p* = 0.49^a^

^a^Mann–Whitney *U* test

IQR, interquartile range

### Treadmill Training

Cernak et al. [61] conducted a single case study with a non-ambulatory 13-year-old girl with ataxia following a brain haemorrhage and reported functionally meaningful improvements in the Gillette Functional Walking Scale (from an initial score of 2 to a final score of 6—walks for household distances) and the WeeFIM mobility and transfer subscales. The intervention consisted of partial body weight-support treadmill training (in conjunction with over-ground walking practice) completed initially in the clinic setting (five days a week for four weeks) and then continued daily at home for further four months (five days a week).

### Hippotherapy

Frank et al. [63] reported short-term (eight weeks) and intermediate (two months) gains in GMFM dimensions D and E in a 6-year-old girl with mild ataxic cerebral palsy (GMFCS I—walks independently with limitations in speed, balance and coordination) following an eight week course of hippotherapy (16 sessions). Gains in the GMFM and the PODCI for global function, sports and physical function, and upper extremity and physical function exceeded the MCID for large effect sizes as interpreted by Oeffinger et al. [73]

### Lycra Garments

Nicholson et al. [52] conducted a before and after study to investigate the effectiveness of wearing a Lycra garment (seven days a week, for six hours, for six weeks) and usual care (physical therapy home programme) on impairment and activity limitations with twelve children with CP, one of whom had ataxia and whose results were reported separately. The PEDI score (activity and participation levels) for this eight-year-old boy improved in self-care, mobility and social domains following completion of the intervention at six weeks. Improvements in proximal stability were reported, but the child was unable to crawl whilst wearing the suit and found it uncomfortable.

## Discussion

The purpose of this systematic review was to evaluate the effectiveness of exercise and physical therapy interventions for children with ataxia. We also aimed to report the type, range, scope and scientific quality of relevant studies. Twenty studies involving 40 children with ataxia met the inclusion criteria. Nine studies included children with ataxia along with children with a number of other primary impairments/diagnoses or grouped children with adult participants. Data for the children with ataxia were unable to be extracted from these studies. The eleven remaining studies provided data from a total of 21 children with ataxia that could be extracted for this review. Our results suggest that only a small number of studies involving a very small number of children with ataxia have been undertaken to investigate the effectiveness of exercise and physical therapy interventions for this population. The lack of RCTs suggests that research for children with ataxia is less well-developed than that for adults. Given that ataxia is a common childhood movement disorder [1] and exercise and physical therapy interventions are the mainstay of treatments available to these children [7], this result may be considered surprising.

The group of eleven studies considered in the main results for this review were of low methodological quality, consisting principally of single case reports and SCEDs. Overall, inconsistent descriptions and measurement of ataxia, poor reporting of adverse events, lack of long-term follow-up and the significant heterogeneity demonstrated in the type of intervention, age range, functional capacity, outcome measures and the duration, frequency, intensity and setting of the intervention limit the extent to which comparisons can be made across studies. Methodological and reporting limitations reduce the confidence with which conclusions can be drawn about the effectiveness of exercise and physical therapy interventions for children with ataxia. It was also observed that measures of fidelity were poorly reported thus making it difficult to understand if the interventions were practicable, acceptable to the children and their parents and able to be followed as intended.

This systematic review has revealed that research about the effectiveness of physical therapy and exercise interventions for children with ataxia is in a very early phase of its development and currently offers inadequate guidance about the efficacy of exercise and physical therapy interventions for children with ataxia. Nonetheless, the results of the studies reported here were on the whole promising and indicate that outcomes for this population have the potential to be improved through physical therapy and exercise. However, no firm conclusions could be drawn and no recommendations could be made based on the evidence reviewed. If the potential of these interventions is to be realised, stronger research designs that counter the limitations of the studies undertaken to date will be needed.

RCTs would make an important contribution to future research. However, recruitment issues and achieving relatively homogeneous samples may challenge the feasibility of running studies of sufficient size. Multi-centre studies and international collaboration might be needed to make these large-scale trials feasible. As an important first step, feasibility trials should be conducted before running fully powered RCTs. This would ensure that all the parts that make up the trial, including recruitment, randomisation, outcome measurement, adherence and compliance, proceed as intended [75] and are acceptable to the children and parents involved. Home-based training, for example, is likely to form a significant component of exercise interventions for children with ataxia [7]; however, Maring et al. [76] reported that although 73% of children with FRA were prescribed a home exercise programme, only 9% of these children carried out the programme as directed. An understanding of the acceptability of, and compliance with, interventions, over the short- and long-term, is critical to the development of RCTs. Potential problems with these programme components could be ironed out not only through running feasibility studies but also by involving children and parents in the design and planning of future studies and intervention programmes. SCEDs and *n*-of-1 trials, including prospective multiple cross-over and randomised case series designs, also offer valid alternatives to RCTs in situations challenged by heterogeneity and when large samples may be difficult to obtain [77]. Clear reporting of, for example, randomisation, primary outcomes, adverse events and blinding of assessors, should be included, and the CONSORT extension for reporting of *n*-of-1 trials [69] should be followed.

Children with ataxia may respond differently to physical therapy and exercise interventions when compared to children with other primary impairments [7] and when compared to adults with ataxia [64]. Involving children with different primary impairments (ataxia, spasticity, athetosis) or combining the data of children and adults in the same study, evident in twelve of the twenty trials that met the inclusion criteria for this review, should be reconsidered unless the potential effectiveness of the proposed intervention can be justified for all participants. If combining children with different pathologies and primary impairments in trials is considered a valid means of testing the efficacy of interventions, future studies should consider involving larger numbers of children with ataxia to enable a separate analysis to be undertaken so that conclusions can be drawn about the impact of interventions for particular groups. Conducting separate studies for children with ataxia arising from a progressive condition to those for children with ataxia arising from a non-progressive form of ataxia seems reasonable, given the likely differing aims of the study, the different underlying pathological mechanisms that could affect the type of intervention used and the expected direction and meaning of responses to interventions. For example, the response to exercise interventions may differ for children with malignant posterior fossa tumours depending on the degree of damage to the dentate nuclei and the inferior vermis [78]. Children with conditions where lesions may be quite discrete are also likely to respond differently to exercise and physical therapy interventions when compared to children with more widespread involvement of the cerebellum, such as that found in progressive conditions [7]. These points stress not only the value of consistent and clear reporting of imaging results and lesion location in intervention studies but also the importance of giving further consideration to the length of follow-up and justifying the recommended dose. Key morbidities, e.g. visual and cognitive impairment, as well as measures of extracerebellar involvement (e.g. via the Inventory of Non-Ataxia Symptoms [79]), should also be reported to offer a more rounded account of a child’s other impairments and a better understanding of the feasibility of delivering the intervention.

This review identified a diverse array of treatment interventions, with regard to the type, intensity, frequency, duration and setting. No justification was provided regarding decisions about dose. Although interventions are tailored according to individual need, these variations make it difficult to compare studies, to carry out meta-analyses, and to conduct replication studies. It is also difficult to examine the effect of usual care as well as other activities that children engage in as details are not always provided, and usual care may include, for example, strengthening, task-specific training, proximal control, balance and stretching exercises. This situation probably reflects the developing but incomplete scientific frame of reference underpinning exercise and physical therapy interventions for people with ataxia [7]. The broad range of interventions and the wide variation in dose, provided in all studies included in this review, may also reflect the lack of consensus about the best approach to take in this field of research. The high number of interventions using some form of technology in studies included in this review (seven studies published since 2012 used video game or virtual reality training programmes) also possibly highlights the potential for technology to drive interventions. These interventions usually include a home training programme which reduces the burden of attending hospital appointments and enables the intense and long-term training that might be necessary to achieve beneficial outcomes [7].

Rehabilitation is targeted at motor learning and adaptation, but it is not clearly understood if individuals with cerebellar dysfunction show similar learning-dependent neuroplasticity to that demonstrated in other areas of the injured brain. A greater understanding of neuroplasticity would provide a firmer foundation for developing exercise and physical therapy interventions to improve outcomes [80]. For example, future studies of exercise and physical therapy for children with ataxia would benefit from including brain imaging to help determine how the brain responds to training protocols of different intensities and may indicate whether neuroplastic changes occur in the cerebellum and/or other parts of the brain [7]. The results of these studies may help to tailor interventions by offering an understanding of the relationship between beneficial outcomes and the frequency, intensity and duration of the intervention. It would also be important to determine, for example, whether positive responses to interventions are related to improvements in ataxia-specific impairments or other training effects, such as improved strength or cardiovascular endurance and/or reduced pain, fatigue or falls, which were rarely measured in the studies included in this review.

Over forty different outcome measures were used in the twenty studies included in this review. The majority of measures focussed on balance and walking and gross motor function. Ataxia severity, dexterity and coordination were rarely reported. Some measures were reported as valid and reliable for children with ataxia. Only one study reported participation-level outcomes (PODCI and PSPCSAYC), and two studies used the PEDI which straddles activity and participation domains. A core set of standardised, valid and reliable measures operating at the impairment, activity and participation levels should be developed for future studies and to facilitate meta-analyses and should be incorporated into a reference group of agreed measures. The SARA and BARS are valid and reliable measures for determining the severity of ataxia in children with posterior fossa tumours [81], and paediatric normative values for the SARA are available [82]. A wide range of valid and reliable participation and well-being measures for paediatric healthcare have been developed (see, for example, Deighton et al. [83]) and should be incorporated into core sets. Data that has established norms for the progression of FRA is also now available (e.g. Friedman et al. [84]) and can be used for comparison to measure the effectiveness of interventions over the long-term and for calculating sample sizes.

## Limitations

A comprehensive literature search was undertaken to identify studies concerning physical therapy and exercise interventions for children with ataxia. Although it is possible that some papers may have been missed, the search was wide-ranging and identified all the studies involving children reported in other reviews and additional studies that had not been previously reviewed. Full text screening was undertaken for a significant number of papers, as reported in Fig. 1, as it was not clear, through title and abstract screening, whether children were participants. Clearer use of indexing and key words would therefore be of value to more easily identify studies for future systematic reviews as research in this field grows. As discussed in the results, we were unable to extract data from studies with mixed populations as the results from participants with ataxia were not reported separately. This meant that the overall reporting of results refers to a small number of children; however, this does reflect the limited number of studies with homogeneous patient groups and the small number of studies undertaken to date with children with ataxia.

## Conclusions

This paper provides an up-to-date review of the literature regarding physical therapy and exercise interventions for children with ataxia. The results highlight the lack of rigorous research undertaken to date for this population, despite physical therapy interventions being a mainstay of treatment for this group of children. Key limitations of the reviewed studies included the following: small participant numbers, low methodological quality, heterogeneity in the nature of the populations and outcome measures used and lack of long-term follow-up. Positive short-term trends were reported in the reviewed studies, suggesting the tested interventions have potential therapeutic value. However, it is not possible to make formal recommendations for clinical practice based on the findings of this review.

The results of this systematic review indicate that high-quality, child-focussed studies are urgently needed. Results from RCTs with adults are not directly applicable to children, which adds impetus to the need to carry out further research with children. Ataxia significantly impacts children’s access to education and participation in everyday activities and future life opportunities; it is, therefore, important to consider what would constitute optimal physical therapy-led interventions for this population.

Intervention studies should draw on theoretical principles, experimental neuroscience and motor-learning studies and other practical observations of what is likely to work in children with cerebellar damage. Feasibility studies should be undertaken before engaging in full-scale RCTs. Well-designed SCEDs with small groups of children may also help to test possible interventions and delivery configurations and would produce outcome measure data that could inform larger trials. Further attention to the development and testing of existing outcome measures for children, as well as consensus agreements about which measures should be used, would also strengthen trial design and facilitate comparisons across studies. Quality of life and participation measures should be recognised as a fundamental requirement. Where possible, imaging results should be reported. Parents and children should be involved in study design, and interventions (including type and delivery dose, as well as fidelity to protocols) should be clearly reported to allow efficacy and effectiveness to be determined. Multi-centre and international collaboration may be necessary to recruit sufficiently large samples for RCTs.

## Electronic supplementary material

ESM 1 (DOCX 16 kb)
