# Supplementary material for: Exercise and Physical Therapy Interventions for Children with Ataxia: A Systematic Review
Source: Cerebellum. 2019 Aug 7;18(5):951–68. doi: 10.1007/s12311-019-01063-z (PMC6761087; doi:10.1007/s12311-019-01063-z)
Supplement: Supplementary file 1 — (DOCX 16 kb) [file 12311_2019_1063_MOESM1_ESM.docx]

**Appendix 1. Data Extraction Checklist**

- Authors, date of publication
- Title
- Journal, volume, issue, page numbers
- Abstract
- Location of study (country)
- Research ethics approval
- Study design
- Where were participants recruited from?
- Method of recruitment?
- Sample size; total n randomised, n for control group and n for experimental group
- Baseline imbalances
- Withdrawals and exclusions after randomisation
- Study population: age, sex, ethnicity, diagnosis (type)
- Duration of condition
- Inclusion criteria
- Exclusion criteria
- Ability to walk, upper limb function (report classification scales and measurement data where available)
- Intervention: aim
- Intervention: type e.g. conventional PT (describe); aerobic, resistance etc.
- Setting for the intervention and special equipment required
- Supervised or unsupervised
- Exercise type: e.g. strengthening/resistance training, endurance, CV fitness, co-ordination, dexterity, balance, posture, functional training
- Exercise mode e.g. treadmill, ergometer
- Co-interventions (not part of the intervention but provided at the same time)
- Duration of the intervention
- Dose (minutes per session / no of sessions per week)
- Tailoring the intervention to participants/modifications to intended intervention
- Intervention provider (e.g. PT, PTA), and special training
- Fidelity / adherence (how assessed and by whom (report degree of adherence where measured.)) Note if not measured/reported.
- Measures of treatment effect (short term 0-1mo post intervention; intermediate 1mo-6mo post intervention; long-term >6mo post intervention)
- Outcomes (list all measures)
- Adverse effects (state what is reported, also if not reported &/or not measured)
- Economic variables (costs)
- Conflicts of interest (state declared CI and list, or state not declared)
- State sources of funding or state not funded or state not declared
- Risk of bias - where applicable complete sheet 2 RoB (details)
- Measures of treatment effect (report results for each outcome for all time points)
- Number of missing participants (compared to number randomised), reasons missing, no. participants moved from other group, reason for move?
- Have important populations been excluded from the study?
- Any further comments
